# More comprehensively measuring quality of life in life-threatening illness: the McGill Quality of Life Questionnaire – Expanded

**DOI:** 10.1186/s12904-019-0473-y

**Published:** 2019-10-31

**Authors:** S. Robin Cohen, Lara B. Russell, Anne Leis, Javad Shahidi, Pat Porterfield, David R. Kuhl, Anne M. Gadermann, Richard Sawatzky

**Affiliations:** 1 Departments of Oncology and Medicine, McGill University, Montréal, Canada; 2 Lady Davis Research Institute of the Jewish General Hospital, Palliative Care Research, room E8.06, 3755 Côte Ste. Catherine Road, Montréal, Québec H3T 1E2 Canada; 3 Centre for Health Evaluation and Outcomes Sciences, Providence Health Care Research Institute, Vancouver, British Columbia Canada; 4 Department of Community Health & Epidemiology, College of Medicine, University of Saskatchewan, Saskatoon, Saskatchewan Canada; 5 Daiichi Sankyo Inc, Basking Ridge, NJ USA; 6 Retired RN, MSN, Vancouver, Canada; 7 Departments of Family Practice and Urologic Sciences, University of British Columbia, Vancouver, British Columbia Canada; 8 Human Early Learning Partnership, School of Population and Public Health, University of British Columbia, Vancouver, Canada; 9 School of Nursing, Trinity Western University, Langley, British Columbia Canada

**Keywords:** Quality of life, Measurement, Psychometrics, End of life, Chronic disease, Palliative care, Burden of illness, Chronic illness

## Abstract

**Background:**

Domains other than those commonly measured (physical, psychological, social, and sometimes existential/spiritual) are important to the quality of life of people with life-threatening illness. The *McGill Quality of Life Questionnaire (MQOL) – Revised* measures the four common domains. The aim of this study was to create a psychometrically sound instrument, *MQOL – Expanded*, to comprehensively measure quality of life by adding to MQOL-Revised the domains of cognition, healthcare, environment, (feeling like a) burden, and possibly, finance.

**Methods:**

Confirmatory factor analyses were conducted on three datasets to ascertain whether seven new items belonged with existing MQOL-Revised domains, whether good model fit was obtained with their addition as five separate domains to MQOL-Revised, and whether a second-order factor representing overall quality of life was present. People with life-threatening illnesses (mainly cancer) or aged > 80 were recruited from 15 healthcare sites in seven Canadian provinces. Settings included: palliative home care and inpatient units; acute care units; oncology outpatient clinics.

**Results:**

Good model fit was obtained when adding each of the five domains separately to MQOL-Revised and for the nine correlated domains. Fit was acceptable for a second-order factor model. The financial domain was removed because of low importance. The resulting MQOL-Expanded is a 21-item instrument with eight domains (fit of eight correlated domains: Comparative Fit Index = .96; Root Mean Square Error of Approximation = .033).

**Conclusions:**

MQOL-Expanded builds on MQOL-Revised to more comprehensively measure the quality of life of people with life-threatening illness. Our analyses provide validity evidence for the MQOL-Expanded domain and summary scores; the need for further validation research is discussed. Use of MQOL-Expanded will enable a more holistic understanding of the quality of life of people with a life-threatening illness and the impact of treatments and interventions upon it. It will allow for a better understanding of less commonly assessed but important life domains (cognition, healthcare, environment, feeling like a burden) and their relationship to the more commonly assessed domains (physical, psychological, social, existential/spiritual).

## Background

A crucial starting point for the validity of any instrument measuring quality of life (QOL) is that it assesses the domains important to the population whose QOL it is designed to measure. Creating QOL instruments requires a balance between comprehensive coverage of what is important to the QOL of the population and feasibility of completion, since there are many potentially relevant life domains, and each has many aspects. Feasibility depends in part on where the respondent is in the disease trajectory. QOL instruments that might be used in the last few months of life necessarily need to be brief. When QOL is being measured alongside other variables, it may be necessary to measure only the domains most strongly related to QOL, especially when QOL is not the primary construct of interest. However, when QOL is the primary outcome, or it is feasible to have respondents complete a longer but still brief QOL questionnaire along with collection of other necessary data, it is important that the QOL measure be as comprehensive as possible. Domains widely accepted as essential for measuring the QOL of people who are ill include the physical, psychological/mental, and social – corresponding to the definition of health in the World Health Organization’s constitution [1]. For people with a life-limiting illness, the existential or spiritual domain is increasingly considered essential as well [2, 3].

The McGill Quality of Life Questionnaire (MQOL), first published in 1996, was developed to measure the QOL (subjective well-being) of people with a life-threatening illness throughout the illness trajectory. It assesses the four essential domains for this population: physical, psychological, support, and existential or spiritual (plus one global QOL item) [4, 5]. It has been widely used, in part because of its perceived high content validity and acceptability for people at the end of life [6, 7]. MQOL does not include items on the intensity of a variety of physical symptoms (for which a symptom measure can be used) but rather focuses on the impact of physical symptoms on QOL. Initial selection of MQOL’s content was based on a review of pre-existing QOL instruments for people with cancer, the QOL literature at the time, clinical expertise, and understanding gained through comments patients made to S.R.C. in a study using other QOL instruments. Since QOL is subjective, content is ideally informed by the people whose QOL is to be measured. To improve MQOL, this step was undertaken by Cohen and Leis [8] in a qualitative study to learn from people at the end of life what is important to their QOL. That study revealed that MQOL is missing some domains important for a comprehensive QOL assessment. In addition to the original MQOL domains (physical, psychological, social, and existential), the person’s environment (e.g. privacy; quiet; access to nature; care setting), cognitive functioning, the quality of healthcare, and a sense of being a burden are important to QOL at the end of life. Therefore, a program of research was undertaken to develop a more comprehensive instrument, MQOL-Expanded (MQOL-E), that includes all eight domains.

Several other published studies provide evidence as to which life domains are relevant to the QOL of people with a life-limiting illness (see McCaffrey et al. [3] for some examples). The findings across studies are not completely consistent: different domains are described in different papers, and what appear to be similar concepts are included in domains of different names in different articles. Therefore, in developing MQOL-E, we needed to make decisions about what concepts should be included. There were two primary considerations. 1) To include all domains found to be important in the Cohen and Leis study [8], since that study was the stimulus for the development of MQOL-E. 2) To keep MQOL-E brief enough to be feasible to complete by people who are in the last weeks of life. To keep it brief, we needed to select general content to reflect each domain, rather than include all specific aspects of each domain. The Cohen and Leis study is a good basis for content development because it is one of only two studies to report all eight domains found in a recent systematic review of important aspects of QOL as reported by palliative care patients. The eight domains described in that review are: physical; personal autonomy; emotional; social; spiritual (environment is included here); cognitive; healthcare; and preparatory (for death or to aid family afterward).^1^ [3] Some participants in the Cohen and Leis study mentioned that worrying about finances was stressful, but the data did not suggest elevating this to domain status. Since this theme focused on worry and stress, it was considered by the investigators to be captured in the anxiety item in MQOL. However, because financial concerns have been increasingly identified as a contributor to the QOL of people receiving palliative care [9–12], an item was developed and tested in one of the three datasets informing this paper to assess the extent to which it contributes to the concept of overall QOL [13]. There have been reports describing other issues that might be included in a QOL instrument for people with life-threatening illness [9, 11, 14–18], but other than the financial domain, none have as much support as the eight domains we selected to be included in MQOL-E.

The studies to create MQOL-E also provided an opportunity to improve MQOL by addressing issues that became apparent with its use over time. If possible, we also wanted to reduce the number of items measuring the original four domains, to keep completion feasible despite the domains to be added for MQOL-E. We created a revised questionnaire, MQOL-Revised (MQOL-R) [19] onto which MQOL-E’s new content is added, thus allowing comparison of the original four domains across future studies using either MQOL-R or MQOL-E.

This paper reports on the creation of MQOL-E, an instrument to more comprehensively measure the domains relevant to the QOL of people with a life-threatening illness. The aim was to add new subscales for the environment, cognition, healthcare, and a feeling of being a burden to MQOL-R’s subscales (Physical, Psychological, Existential, Social), and to test the relevance of the financial domain to the concept of overall QOL.

## Methods

### Item development for the new domains

SRC, AL, and the late Terry Bunston, PhD, with feedback from a larger research team, developed initial items to capture three new domains (Environment; Cognition; Healthcare) and the construct of (feeling like a) Burden. These were combined with select MQOL items to create a 32-item questionnaire. Like MQOL and MQOL-R, each MQOL-E item has a 2-day timeframe. Sixty palliative care patients from inpatient and home care settings in three Canadian cities and 10 palliative care clinicians then rated each item for importance to patient QOL (0–10 scale) and indicated items they found unclear (criterion of ≤10% for retention), redundant, or upsetting. For example, an earlier item stem, “I feel like a burden”, was upsetting and unclear (“burden to whom?”) to some participants, but they deemed the content important. It was revised to “I felt badly about how my situation affected the people I care about”. Rejected items were replaced. The new items were tested with another 63 patients, who completed the revised 26-item questionnaire to evaluate item distribution and again assess clarity and redundancy. New revisions resulted in a 20-item questionnaire. A validation study with this new questionnaire found the factor structure to be unsatisfactory since items representing different domains did not load as predicted (*N* = 304; unpublished). Various new changes and 11 experimental items, including one for the new financial domain, were explored in 23–27 item questionnaires in the three studies that provided datasets for this report. Since other instruments were completed at the same time in each of these studies [13, 20], to respect the physical condition of the participants, not all potential replacement items could be tested at once. To create the domain of quality of healthcare, Study G (the chronologically earliest dataset used in this report) used the two items from the immediately preceding 20-item version and also tested three new items, since we were hoping to create items with improved distributions. The new items, which did not have substantially improved distributions, were not included in subsequent studies. Only the two older items, which had been reviewed by patients during the development of the 20-item version and for which more data were available (since they were used in studies E and F), were included in the present analyses.

Items were initially developed in English, then translated into French by a professional translator. These two versions were compared by bilingual patients and clinicians; slight discrepancies between the two versions were reconciled. Sometimes this required a different word in French, other times the French translation suggested that the English wording needed clarifying, which was done. These changes did not affect the initial intent of the item. The resulting French version was successfully professionally back-translated into English with no further changes required. Both language versions were used in collecting datasets E and G, where participants chose the language version they preferred. Study F used only the English version since it was part of a larger study conducted in English in a city where English is the language of daily interaction.

### Items used in tests of construct validity

To develop the final version of MQOL-E, we tested the seven new items representing the new domains and burden construct described in the Introduction and shown in Table 1 (the original 11 minus the four for quality of healthcare that were removed as explained above). As with MQOL-R, all items are measured on an 11-point scale from 0 to 10, anchored as indicated.

**Table 1:** Domains and corresponding items tested for inclusion in MQOL-E

Domain	Item stem [time frame of past 2 days (48 h) not shown]	Item end anchors
Healthcare	Getting the information I needed from the health care team was: The quality of health care I received was:	difficult/very easy unsatisfactory/extremely good
Cognitive functioning	I was able to think clearly: My memory worked:	not often/always very poorly/very well
(Feeling like a) Burden	I felt badly about how my situation affected the people I care about:	not at all/completely
Environment	My physical surroundings met my needs	not at all/completely
Financial	My financial situation has been stressful	not at all/completely

### Datasets, participants and consent

The datasets used in the analyses reported here (E-G) were collected from people with life-threatening illnesses at all stages of the disease trajectory or aged > 80 years. They were recruited from 15 Canadian healthcare sites in seven provinces. Study E (*N* = 219) was designed to test the construct validity of a 24-item pre-cursor instrument to MQOL-E [13]. The primary purpose of Study F (*N* = 368) was to develop a self-report instrument to measure satisfaction with healthcare. A 23-item pre-cursor instrument to MQOL-E was used in that study to assess the construct validity of the satisfaction with care instrument [20]. Study G (*N* = 216) used a 27-item pre-cursor to MQOL-E in a longitudinal study of the QOL of palliative care patients and was also intended to be used to assess construct validity (unpublished). Table 2 provides demographic information about the participants in each study. Datasets E and G include only people with cancer, while F includes people with various end-stage diseases and/or advanced age. Participants were recruited from a range of settings: palliative home care and inpatient units, acute care units, and oncology outpatient clinics. In studies F and G, the mean age was over 65; similar numbers of women and men participated. Study E recruited from oncology clinics, where the participants were somewhat younger (mean 58 years) and 64% were women. More detailed information about the three study samples is provided in Table 2.

**Table 2:** Demographics for each sample used in the development of MQOL-E

Variables	Study^a^		
	E	F^c^	G^d^
N	219	368	216
Gender (% female)	64	48	51
Age (mean; standard deviation)	58 (15.4)	77 (9.9)	66 (12.5)
Highest level of education (%)^b^			
Did not complete high school	10	46	32
High school completed but no post-secondary	34	22	20
Some post-secondary	56	30	42
Disease	Cancer	Various end-stage diseases (COPD; CHF; cirrhosis; cancer) or > 80 years old	Cancer
Settings (%)	Outpatient clinics (100)	Acute care units (71) Home care (22) Palliative care units (7)	Palliative care units (71) Palliative home care (27) Other (1) Missing (1)

^a^Since these studies were also used in development of MQOL-R, the same naming convention was used [19].

^b^For Study E, participants reported the highest level of education attended. For other datasets, participants reported the highest level of education completed. ^c^ Timeframe of 1 month rather than 2 days. ^d^ Longitudinal study. The first questionnaire completed by each participant was selected to provide the largest n (i.e. before dropout)

In studies F and G, written informed consent was provided; participants had the choice of completing the questionnaire on their own or as a structured interview. In study E, with permission of the Research Ethics Committee, potential participants were provided with an information sheet and questionnaire to be completed and returned anonymously; consent was implied if the questionnaire was completed. All of the studies for item development and the current analysis were approved by the research ethics boards of all of the institutions from which patients were recruited.

### Analysis

Candidate items and domains were tested as additions to MQOL-R in four steps by conducting Confirmatory Factor Analyses (CFAs) using maximum likelihood robust estimation. The procedures, criteria for determining model fit, and goal of each step are described in Table 3. Analyses were conducted using the Mplus software [21]. Model fit was assessed via the Comparative Fit Index (CFI; >.95 indicates good fit) and the Root Mean Square Error of Approximation (RMSEA; <.06 indicates good fit) using criteria from Hu and Bentler [22]. The chi-square test was not used as it is sensitive to sample size [23]. To ensure that the items intended to represent new domains represented a construct different from existing MQOL-R domains, we first tested the factor loading of each item on existing domains. A standardized factor loading ≥ .6 was considered to form part of an existing domain. This is higher than the usual minimum criterion of ≥ .4 [24] because these items were designed to measure different aspects of QOL than the MQOL-R domains, therefore, adding them to an existing domain instead would require strong theoretical support. Nevertheless, items with loadings between 0.4 and 0.6 were closely scrutinized for conceptual fit on existing MQOL-R domains. To assess whether it is justified to create a summary score of all of the domains, the usual criterion of a factor loading ≥0.4 was used. Missing data were accommodated via full information maximum likelihood estimation. The total percentage of missing data was 18% when including all variables and 13% when including the variables in the final model.

**Table 3:** Analysis methods and results

Step	Goal of analysis	Procedure and criteria for model and item fit	Results	Conclusions
1	To ensure that new items, which were conceived to represent new domains, did not reflect existing MQOL-R domains	Each of 7 candidate items (see Table 2) was added one at a time to the MQOL-R model and allowed to cross-load on all existing domains of the MQOL-R *Criteria to conclude the item belongs to an existing MQOL-R domain* • Comparative Fit Index (CFI) > 0.95 • Root Mean Square Error of Approximation (RMSEA) < 0.06 • Standardized factor loadings ≥0.6 on existing domains. Loadings between .4 and .6 were examined for conceptual fit with existing domain	Two items had standardized factor loadings > 0.4 and < 0.6: • FINANCE on the Psychological domain (0.47) • BURDEN on the Physical domain (0.49)	BURDEN: evidence from the qualitative interviews had suggested that this is a separate aspect of QOL from the physical domain. In addition, the loading did not reach the criterion of ≥0.6 for addition to a domain. BURDEN was therefore not added to the Physical domain FINANCE: Conceptually different from psychological symptoms. May have loaded with them because the item captures stress due to financial situation. In addition, the loading did not reach the criterion of ≥0.6 for addition to a domain. FINANCE was therefore not added to the Psychological domain
2	Determine if each new domain individually fit with MQOL-R	The 5 new domains (see Table 2) were added to the MQOL-R model one domain at a time (items were assigned to the new domains for which they were designed). For new domains that comprised a single item, the factor loading was fixed at 1.00 and the residual variance was fixed at zero. *Criteria* • CFI > 0.95 • RMSEA < 0.06 • Standardized factor loadings for all new items should be ≥ to all loadings for that item in Step 1	• CFI range: 0.96–0.97 • RMSEA range: 0.035–0.041 • Standardized factor loadings for all items for new domains were higher than in Step 1 (range: 0.72–0.88)^a^	All new domains fit with the existing MQOL-R, and were retained for step 3
3	Assess construct validity. Establish fit of CFA model of final version of MQOL-E	Domains retained in the separate analyses in Step 2 were added all at once to MQOL-R. Another model was fit without the Finance domain *Criteria* • CFI > 0.95 • RMSEA < 0.06	Model with Finance domain • CFI = 0.97 • RMSEA = 0.033 • Standardized factor loading range: 0.53–0.91^b^ Model without Finance domain • CFI = 0.96 • RMSEA = 0.033 • Standardized factor loading range: 0.52–0.91^b^	The model with new domains had good fit, both with and without the Finance domain, providing support for the addition of the new domains
4	Assess whether MQOL-E domains measure a single underlying construct (overall QOL), which would support the calculation of a summary score. If so, examine the relationship of the summary score to a single item measuring global QOL Assess importance of the Finance domain to overall QOL	The model of MQOL-E from Step 3 was fit with the addition of a second-order factor for overall QOL. This was repeated with the Finance domain removed. The correlation of the overall factor with a single-item scale (MQOL-SIS) measure of global QOL was calculated. *Criteria* • CFI > 0.95 • RMSEA < 0.06	Model with Finance domain • CFI = 0.93 • RMSEA = 0.042 • Standardized factor loading of Finance domain on second-order factor: 0.25 Model without Finance domain • CFI = 0.93 • RMSEA = 0.043 • Standardized factor loadings for both first and second order factors are shown in Fig. 1 • Correlations of the factors with the MQOL-SIS are reported in Table 4 Domain means, standard deviations and internal consistency estimates (Cronbach alphas) are reported in Table 5	The second-order MQOL-E model with the Finance domain had overall acceptable model fit; although the CFI was slightly below 0.95, the RMSEA clearly meets the criterion for good fit. The loading of the Finance domain on the second-order factor was very low. The models with and without this domain had essentially the same fit (RMSEA difference of 0.001 and identical CFI). Since the Finance domain appeared to be a weak indicator of overall QOL and removing it did not result in poor model fit, the model without this domain was retained. This reduces the number of items and thus reduces participant burden.

^a^The Burden, Environment, and Finance factors were each represented by a single item. The loadings of these items were therefore fixed at 1.00; comparisons with Step 1 are not relevant

^b^This does not include the loadings for the single-item Burden, Environment and Finance factors since these loadings were fixed at 1.00

Although McCaffrey et al (2016) included the concept of ‘feeling like a burden’ in the social domain (as did Cohen and Leis [8]), we were not certain whether it belonged in the social, psychological, or existential domain, or whether it would need to represent a separate domain. Assessment of this during creation of MQOL-R indicated it did not load sufficiently on any of the MQOL-R factors to justify its inclusion on any original domain, therefore it was included in the MQOL-E analyses as a separate factor. To assess the relevance of the financial domain, models both with and without the financial domain item were tested to determine the extent to which it made a unique contribution to the measurement of QOL.

MQOL-E subscale scores are calculated as the mean of the items measuring that domain. The MQOL-E summary score is the mean of the subscale scores (it does not include the global item). The Pearson correlations between the latent domains, second order factor, and a global measure of QOL were calculated for descriptive purposes. Cronbach’s alpha (α) was calculated to assess internal consistency reliability; an alpha ≥0.7 is considered acceptable [25].

## Results

The results and conclusions of each step of the analyses are described in Table 3. None of the new items loaded ≥0.6 on the MQOL-R factors, suggesting that they could represent new domains. There was good model fit when each of the 5 new domains was added separately to MQOL-R, as well as when all new domains were added together (CFI = 0.97; RMSEA = 0.033), supporting the correlated factor structure of MQOL-E. There was acceptable model fit for a second-order factor, supporting the creation of a MQOL-E summary score (CFI = 0.93; RMSEA = 0.042). However, the financial domain was removed from the final MQOL-E because the standardized loading on the second-order factor was low (0.254, well below the 0.4 criterion) and its removal had little effect on the CFI and RMSEA of the first-order (CFI = 0.96; RMSEA = 0.033) and second-order (CFI = 0.93, RMSEA = 0.043) models. This suggests that the Finance domain is a weak indicator of overall QOL. In keeping with our goal of ensuring MQOL-E is as brief as possible, the model without this domain was retained.

**Fig. 1:**
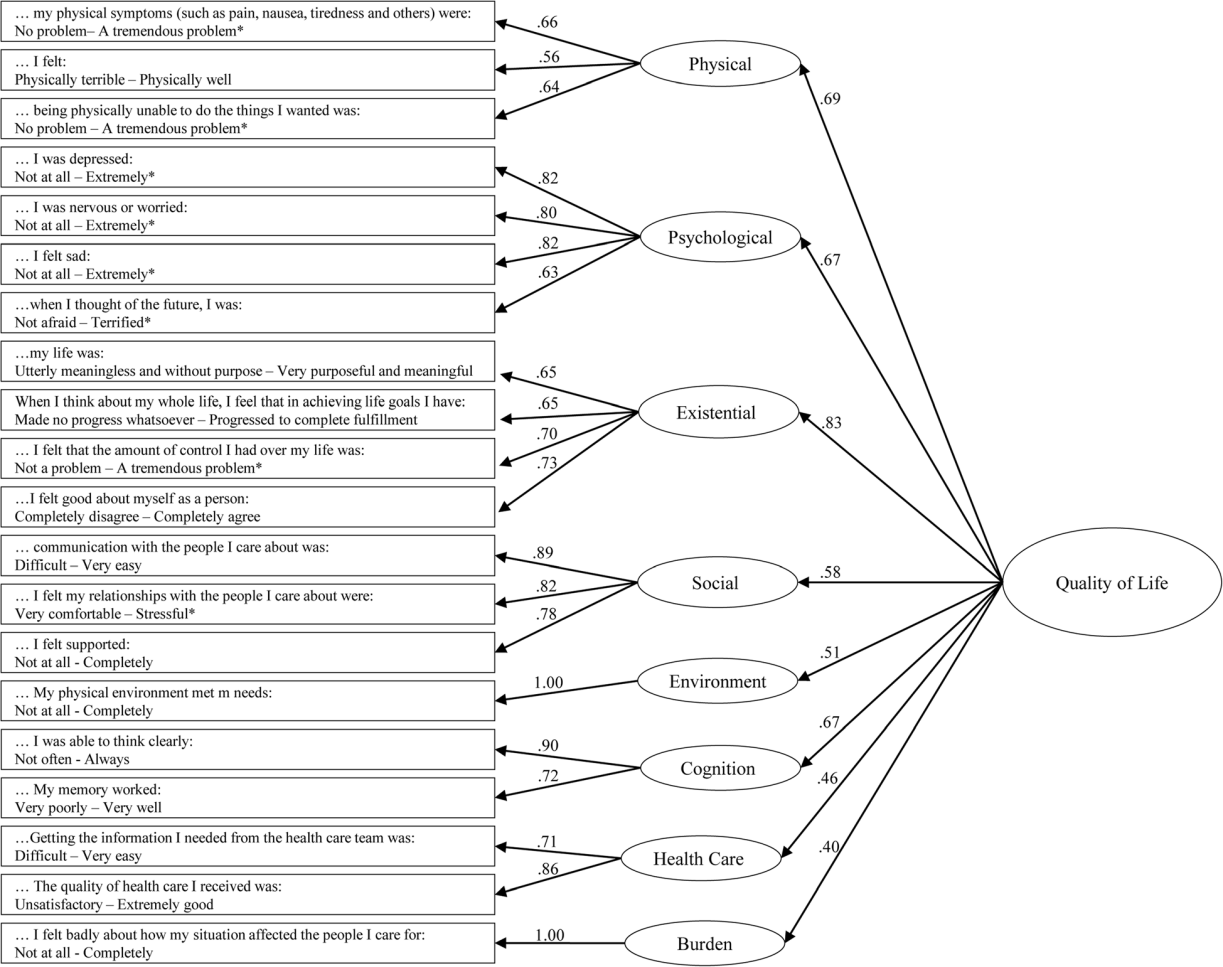
MQOL-E items and CFA for first (subscale) and second order (overall QOL) latent factors. Numbers indicate standardized factor loadings. * = reverse scored items. … = Over the past 2 days (48 h). Model fit statistics: RMSEA: 0.043; CFI: 0.932

The items and standardized factor loadings for the final model are shown in Fig. 1. For the second-order factor, factor loadings ranged from 0.40 for the Burden domain to .83 for the Existential domain.

Domain means, standard deviations, and Cronbach's alphas are shown in Table 4. Cronbach’s alpha for the domains measured with more than one item for the final model ranged from 0.66 to 0.87 (Physical = 0.66; Psychological = 0.85; Existential = 0.78; Social = .87; Healthcare = 0.76; Cognitive = 0.79). Table 5 shows the correlations with the global item (MQOL-SIS) and inter-domain correlations, all of which were statistically significant (*p* < .001) except for the correlation between the Cognitive and Burden domains (*p* > .05). The highest correlation is between the MQOL-SIS and the 2nd order factor (*r* = .65). The correlations between the MQOL-SIS and the domains are between .64 (Physical) and .30 (Social and Burden). The Healthcare domain has similar correlations with all of the other domains (.25–.34) except that it is low for Burden (.16). The Cognitive domain is most highly correlated with the Existential domain (.56). The Burden domain is most highly correlated with the Physical domain (.53). The Environment domain is most highly correlated with the Existential (.44) and Cognitive (.43) domains, with a particularly low correlation with the Burden domain (.07).

**Table 4:** Domain mean scores and standard deviations, Cronbach alpha

Domain	Mean (SD)^a^	Cronbach Alpha
Physical	5.11 (2.48)	0.66
Psychological	6.83 (2.49)	0.85
Existential	7.19 (1.99)	0.78
Social	8.63 (1.78)	0.87
Healthcare	8.56 (1.79)	0.76
Cognition	8.23 (1.97)	0.79
Burden	4.58 (3.59)	N/A^b^
Environment	8.27 (2.16)	N/A^b^

^a^MQOL-E scores range from 0-10, with 0 indicating the poorest QOL and 10 the best

^b^Not applicable because the domain is represented by a single item

**Table 5:** Pearson correlations between the second order factor, latent domains, and the single-item global measure of quality of life (MQOL-SIS)

	MQOL-SIS	Physical	Psycho-logical	Existen-tial	Social	Health-care	Cognitive	Burden
2nd order factor	.65							
1st order factor model								
Physical	.64							
Psychological	.43	.62						
Existential	.52	.46	.57					
Social	.30	.28	.35	.52				
Healthcare	.32	.27	.25	.34	.28			
Cognitive	.31	.36	.41	.56	.45	.34		
Burden	.30	.53	.33	.34	.20	.16	.20	
Environment	.32	.27	.26	.44	.32	.33	.43	.07

## Discussion

This study demonstrates that the four-domain MQOL-R can be expanded to include four new domains that people at the end of life report as important contributors to their QOL [8]. MQOL-E more closely reflects the domains that palliative care patients report are important to their QOL than existing QOL questionnaires while remaining brief. While we believe that MQOL-R is sufficient and appropriate when a shorter measure is needed to reduce response burden, MQOL-E gives a more well-rounded or comprehensive description of the QOL of people with a life-limiting illness. While longer than MQOL-R (15 items) [19], at 21 items MQOL-E can be completed by many people who are seriously or terminally ill when a more comprehensive assessment of QOL is desired, such as when QOL is the primary outcome of interest in a study. Structuring the MQOL-E so that the first 15 items are MQOL-R allows for flexibility, such as comparison to studies using MQOL-R or, when collecting longitudinal data, to shorten the questionnaire for people who become unable to respond to the complete MQOL-E.

Factor analysis supports the creation of a summary score for MQOL-E based on the eight domains. We tested the construct validity of calculating a summary score because it is necessary for many purposes. However, we advise using a profile of the individual subscale scores whenever possible, because they are more informative. For example, some domains may improve over time while others worsen, resulting in little change over time in the summary score despite important change occurring at the domain level. A strength of MQOL-E is that the factor analysis supports using separate scores for each of the eight domains. MQOL-E therefore provides information about a wider range of QOL contributors than instruments assessing only the physical, psychological, and social domains, as well as those that also include the spiritual or existential domain. The internal consistency of the Physical domain (Cronbach’s alpha = 0.66) was slightly below the accepted criterion of ≥0.7. This may be because this domain is measured by items that reflect quite diverse aspects of physical health: 1) how much of a problem it is not to be able to do things you want to do; 2) how much of a problem physical symptoms are; and 3) a feeling of general physical wellbeing. To keep the instrument brief, each of these concepts is measured with a single item, likely lowering alpha.

The moderate correlation of the MQOL-E summary score with a single item measuring global QOL (*r* = .65) suggests that MQOL-E measures QOL in a way that is related but not identical to a global assessment of QOL. The lower correlations between the global QOL item and the new domains added for MQOL-E underscores the value of measuring these aspects of QOL individually rather than assuming they will be reflected in a global assessment. The moderate correlations between several domains indicate that the experience in one life domain is related to the experience in other domains. The correlations of the new domains with other domains provide valuable new insights. For example, the Burden domain is most strongly correlated with the Physical domain (*r* = .53), suggesting that physical wellbeing is more important than other domains in the extent to which one feels like a burden. However, since people with significant cognitive impairment were not included in the study, it is possible that significant cognitive impairment is also important to feeling like a burden.

There are some limitations to the study. While it is a strength that MQOL-E was created using datasets collected in many cities in various care settings, because these studies used various versions of the questionnaire to test different items, the MQOL-E described in this paper was not tested in its exact final form. Another limitation is that some domains are measured with only one or two items. This may limit the scope of the content covered within each domain as well as its reliability. We imposed this limitation on MQOL-E to keep it brief and facilitate its completion throughout the illness trajectory and as close to the end of life as possible. Most (but not all) people who contributed data to the study had cancer, therefore it is unknown whether the domain structure will be the same when tested with samples with only non-cancer life-limiting illnesses. However, it is noteworthy that McCaffrey et al’s systematic review found that aspects of QOL do not differ according to diagnosis, care setting, or living arrangement [3]. A strength of MQOL-E is that it was developed simultaneously in English and French. However, the potential for differences in the structure of the English and French versions could not be investigated with the present data (i.e. testing for measurement invariance) and should be investigated in future studies. Furthermore, people in the recruitment settings who spoke neither language are not represented in this study. In order to allow QOL instruments to be completed even by people who are quite weak, or have difficulty seeing or are illiterate, it is important that they can be read aloud to the respondent, as well as self-completed by those without these limitations. In this study we combined data from both methods of administration to use data from the widest variety of respondents. Because we did not have enough data to test whether MQOL-E’s structure is the same for different modes of administration, future studies should examine this.

MQOL-E includes seven of the eight domains found by McCaffrey et al to have solid evidence for being important to the QOL of palliative care patients [3]. The domain not covered is preparation for death and afterward. After initially considering including this domain, we later decided to exclude it because preparation is an activity, whereas MQOL-E is intended to measure QOL as a subjective experience. If preparation enhances QOL, we expect that this would be through improving other domains, such as (feeling like a) burden, social, or existential.

As illnesses progress, healthcare takes on an increasingly large role in a person’s life. Measures of the occurrence (or not) of specific aspects of healthcare are usually considered person-centred experience or process measures, while assessments of QOL are considered to be outcome measures. Nevertheless, MQOL-E includes healthcare as a domain of QOL for two reasons. First, healthcare was found to be important to QOL in the Cohen and Leis study [8] on which MQOL-E is based and in 13/24 studies in the McCaffrey et al review [3]. Second, the MQOL-E healthcare items involve a subjective rating of quality, rather than simply the occurrence of aspects of healthcare as found in patient experience instruments.

We created a new domain, Burden. Not only is feeling like a burden important to QOL, it is one of the major reasons that people request a hastened death [26–28], and therefore important in QOL assessment. We initially expected this item to load on the social domain, but it loaded moderately on the physical domain. We believe this is so because the worse their physical condition, the more help the respondent is likely to need from others, potentially leading them to feel more like a burden. McCaffrey et al [3] note this relationship in describing the personal autonomy domain, but also mention the concept of burden in describing the preparatory (taking care of one’s affairs is perceived to relieve burden on family) and social domains. While relevant to several domains, this item was kept as a separate one in MQOL-E to ease interpretation, and to maintain the items measuring the physical domain identical to those in MQOL-R.

It is not clear in the literature whether finances are an important contributor to the QOL of people with advanced disease. We considered one item about finances, but ultimately excluded it because it loaded moderately on the psychological domain (possibly because it assessed stressfulness of finances), making it difficult to interpret. If finances are important because they add to feeling like a burden, as has been found in other studies [29], that is captured by our Burden domain. We can only speculate as to why financial concerns were not identified as a distinct domain in measuring overall QOL in our study. It may be that our studies were conducted in Canada, where most healthcare is paid for by the government. Alternatively, as another study in Canada found finances to be a contributor to family caregiver QOL, it may be that many people with very advanced disease leave that concern to their families [30]. A version of MQOL-E including the financial item is available for use when measuring finances as a source of stress is considered important, but this item should not be used in computing the summary score.

The correlations of the new domains with the MQOL-SIS, a global item measuring overall QOL, were .30–.32. These correlations are quite a bit lower than those for the Physical (.64), Psychological (.42), and Existential (.52) domains. However, these correlations are similar to that for the Social domain (.30), which is generally accepted as important to include in QOL instruments [2]. Since the Social domain has a similar correlation but is reported as important by almost all palliative care patients reporting on what is important to their QOL [3], the lower correlations for the new domains may reflect the limitations of measuring QOL with a single global item.

Comparing the domains coverage of MQOL-E to two other widely used QOL measurement systems, the FACIT-PAL (46 items) and its shorter version, FACIT-PAL-14 (14 items) [31, 32], and the EORTC QLQ-C30 and its version for palliative care, the EORTC QLQ-C15-PAL [10, 33], MQOL-E covers more of the domains found in the McCaffrey et al review [3]. The FACIT-PAL comes closest to the domain coverage of MQOL-E, including cognition and feeling like a burden, but it does not cover healthcare or environment. At 46 items it is more than twice as long as MQOL-E. The FACIT-PAL-14 touches upon similar domains to its parent, but unlike the longer version does not include cognition. The EORTC QLQ-C15-PAL touches only the physical, psychological, and personal autonomy domains, while the longer instrument additionally touches on the social and cognition domains but does not cover the spiritual/existential or healthcare domains, nor do they include the concept of burden. None of the instruments covers the preparatory domain, although we do not see that as a drawback, for the reasons explained above. For consideration of some other differences, see Cohen et al. [19].

We are further exploring the psychometric properties of MQOL-E in an ongoing study with a new population, including a confirmatory factor analysis of MQOL-E in its final version, and assessing test-retest reliability and responsiveness to change. Future validation of MQOL-E should include investigation of convergent and discriminant validity and of measurement invariance of the English and French versions as well as mode of administration.

## Conclusion

Given the various life domains that people living with life-threatening illness find important to their QOL, the creation of MQOL-E, by adding the four domains of healthcare, cognitive functioning, (feeling like a) burden, and environment to the four domains covered by MQOL-R (physical, psychological, existential, social), is an important advance in QOL measurement. The statistical confirmation of eight distinct domains is critical since this permits measurement of different aspects of QOL. This study also provides evidence of the validity of a summary score, when desired. MQOL-E is a more comprehensive measure of QOL compared to existing instruments. A strength of the instrument is that it includes MQOL-R, which allows comparison to studies using MQOL-R. We expect that use of MQOL-E will advance our understanding of the well-studied and new facets of the QOL of people with a variety of life-threatening illnesses.

## Supplementary information

**Additional file 1.** Research ethics committees.

## Data Availability

The datasets used and/or analysed during the current study are available from the corresponding author on reasonable request.

## Abbreviations

CFI: Comparative fit index
CHF: Congestive heart failure
COPD: Chronic obstructive pulmonary disease
EORTC QLQ-C15-PAL: European Organization for Research and Treatment of Cancer – Quality of Life Questionnaire – Core15-Palliative Care
EORTC QLQ-C30: European Organization for Research and Treatment of Cancer – Quality of Life Questionnaire – Core30
FACIT-PAL: Functional Assessment of Chronic Illness Therapy – Palliative Care Scale
MQOL SIS: McGill Quality of Life Questionnaire Single Item Scale
MQOL: McGill Quality of Life Questionnaire
MQOL-E: McGill Quality of Life Questionnaire- Expanded
MQOL-R: McGill Quality of Life Questionnaire – Revised
QOL: Quality of life
RMSEA: Root mean square error of approximation

## Footnotes

1 For simplicity, we have subsumed different authors’ specific variations in domain name and content within a single broad domain where there is significant conceptual overlap. For example, despite differences between the existential and spiritual concepts, we have considered them to be one domain. Similarly, we combined the concepts of support, relationships, and social functioning and refer to them as the social domain.
