# Supplementary material for: More comprehensively measuring quality of life in life-threatening illness: the McGill Quality of Life Questionnaire – Expanded
Source: BMC Palliat Care. 2019 Oct 31;18:92. doi: 10.1186/s12904-019-0473-y (PMC6823967; doi:10.1186/s12904-019-0473-y)
Supplement: Supplementary file 1 — Additional file 1. Research ethics committees. [file 12904_2019_473_MOESM1_ESM.pdf]

The following ethics committees and hospitals in Canada approved the original studies that collected the data used in this report. Some do not have study numbers, because some studies were conducted when and where studies were identified by title only or when such detailed information was not required for publication and was therefore not retained.

Dataset E
Biomedical Research Ethics Committee, Jewish General Hospital, Montreal, Quebec #09-084

Dataset F
Queen’s University Health Sciences & Affiliated Teaching Hospitals Research Ethics Board #DMED-1029-07
St. Joseph’s Hospital Research Ethics Board (Hamilton, Ontario)
Dalhousie University Health Sciences Research Ethics Board
University of Alberta Health Research Ethics Board
University of British Columbia Behavioural Research Ethics Board
Comité d'éthique de la recherche, Hôpital Maisonneuve-Rosemont

Dataset G
McGill University Faculty of Medicine Institutional Review Board #A08-B32-01B,

- Research Ethics Committee, Jewish General Hospital (central review by McGill)
- McGill University Health Centre #97-004 (central review by Mcgill)

Sisters of Charity of Ottawa (SCO) Health Service research Ethics Board
The Ottawa Hospital Research Ethics Board #2001503-01H
Queen’s University Health Sciences & Affiliated Teaching Hospitals Research Ethics Board #ONGY-251-04
University of British Columbia Behavioural Research Ethics Board B01-0668
Interior Health, Central Okanagan, Kelowna General Hospital Institutional Research Review Committee #B01-0668
University of Saskatchewan Research Ethics Board (Behavioural) BSC #01-226
